# MicroRNA-146 inhibits pro-inflammatory cytokine secretion through IL-1 receptor-associated kinase 1 in human gingival fibroblasts

**DOI:** 10.1186/1476-9255-10-20

**Published:** 2013-05-16

**Authors:** Yu-Feng Xie, Rong Shu, Shao-Yun Jiang, Da-Li Liu, Jing Ni, Xiu-Li Zhang

**Affiliations:** 1 Department of Periodontology, Ninth People’s Hospital, School of Medicine Shanghai Jiao Tong University, Shanghai Key Laboratory of Stomatology, 639 Zhi Zao Ju Road, Shanghai 200011, China; 2 Shanghai Research Institute of Stomatology, Ninth People’s Hospital, School of Medicine Shanghai Jiao Tong University, Shanghai Key Laboratory of Stomatology, Shanghai, China

**Keywords:** miRNA-146, Periodontitis, Human gingival fibroblasts, Pro-inflammatory cytokines

## Abstract

**Background:**

Although various microRNAs (miRNAs) regulate immune and inflammatory responses, the function of miRNAs in periodontitis has not been clearly illuminated. In this study, we measured miRNA-146 (miRNA-146a and miRNA-146b-5p) expression and explored its regulatory function in the inflammatory response in human gingival fibroblasts (HGFs).

**Methods:**

miRNA-146a and miRNA-146b-5p expression was measured by performing real-time polymerase chain reaction in HGFs after *Porphyromonas gingivalis* (*p.g*) lipopolysaccharide (LPS) stimulation. After the HGFs were transfected with miRNA-146a and miRNA-146b-5p inhibitor, the expression levels of interleukin-1β (IL-1β), interleukin-6 (IL-6) and tumor necrosis factor-α (TNF-α) were measured by enzyme-linked immunosorbent assay (ELISA). Meanwhile, IL-1 receptor-associated kinase 1 (IRAK1) and TNF receptor-associated factor 6 (TRAF6) were detected by western blot and quantitative PCR. A luciferase assay was used to detect whether miRNA-146 could directly bind to the 3’-UTR of IRAK1.

**Results:**

The expression levels of miRNA-146a and miRNA-146b-5p significantly increased in the *P.g* LPS-stimulated HGFs compared to the non-stimulated HGFs. The inhibition of miRNA-146a and miRNA-146b-5p resulted in increased IL-1β, IL-6 and TNF-α secretion. The mRNA and protein levels of IRAK1, but not TRAF6, also increased. We further found that miRNA-146a and miRNA-146b-5p directly bound to the IRAK1 3’-UTR.

**Conclusion:**

Our data suggest that miRNA-146 inhibits pro-inflammatory cytokine secretion through IRAK1 in HGFs, which indicates that miRNA-146 functions as a negative regulator of periodontal inflammation.

## Background

Periodontal disease is induced by a group of pathogenic microorganisms, such as *Porphyromonas gingivalis* (*P.g*), which leads to inflammation and the destruction of periodontal tissues. The innate immune response is the most important line of defense against putative periodontal pathogens and virulence factors, such as *P.g* and *P.g* LPS [1]. Human gingival fibroblasts (HGFs), which are the major components of gingival connective tissue, directly interact with bacteria and bacterial products, including LPS, in periodontitis [2].

microRNAs (miRNAs) are an abundant class of short (20 to 25 nucleotides), non-coding RNA molecules. They function as post-transcriptional regulators that bind to complementary sequences in the 3' untranslated regions (3' UTRs) of target messenger RNA transcripts (mRNAs), usually resulting in gene silencing [3,4]. miRNAs are implicated in establishing and maintaining the cellular fate of immune cells and are involved in innate immunity by regulating Toll-like receptor signalling and ensuing a cytokine response [5]. Recent studies have reported different miRNA expression patterns between healthy tissues and inflamed tissues inflicted with periodontal disease, which indicates that miRNAs may be involved in the regulation of periodontal disease [6,7]. However, the function of miRNAs in HGFs during periodontitis remains unclear. miRNA-146 is composed of miRNA-146a and miRNA-146b-5p. It has been demonstrated that miRNA-146a and miRNA-146b-5p are involved in inflammation in tissues in addition to gingival tissue [8,9]. Recent studies reported that interleukin-1 receptor-associated kinase 1 (IRAK1) and tumor necrosis factor receptor-associated factor 6 (TRAF6) are direct targets of miRNA-146 [10]. Based on these reports, we wondered whether miRNA-146 affected the gingival inflammatory response though IRAK1 and TRAF6.

In this study, we report that miRNA-146a and miRNA-146b-5p are up-regulated in response to *P.g* LPS stimulation in HGFs. We also showed that miRNA-146 inhibition results in an increase in pro-inflammatory cytokines, such as IL-1β, IL-6 and TNF-α, through IRAK1 activation. We found that miRNA-146 inhibits IRAK1 expression by binding directly to the 3’-UTR of IRAK1. Our data suggest that miRNA-146 is a negative regulator of the immune response in periodontal disease.

## Methods

### Primary cell culture of HGFs

Approval for conducting the experiments on the human tissue specimens was obtained from the Committee of Ethics in Research of the School of Medicine, Shanghai Jiao Tong University. After informed consent was obtained from each dental patient undergoing oral surgery, the discarded gingiva was collected. The explants of the gingiva were obtained from 10 patients (4 males and 6 females aged from 26 to 63 years old) who were nonsmokers and did not have any systemic diseases. Apart from periodontal scaling and root planning treatment, the subjects did not receive other treatments or take any prescribed medication. The epithelial tissues were separated from the gingiva after 24 hours of soaking in 2 U/ml of Dispase II (Takara, Japan). The gingival connective tissues were cut into pieces and cultured in Dulbecco’s modified Eagle’s medium (DMEM) (Gibco, USA) supplemented with 20% fetal bovine serum (FBS) (Hyclone, USA) and antibiotics (50 μg/ml of streptomycin sulfate, 100 U/ml of penicillin) [11]. The medium was changed every 3 days for 10–20 days. The cells were passaged when confluent cell monolayers were formed [12].

### RNA extraction

After 4 passages, total RNA was extracted from the HGFs using TRIzol reagent (Invitrogen, USA) according to the manufacturer’s protocol. The RNA was stored at −80°C until further use. For the commercial miRNA microarray analyses (Kangchen Bio-Tech, Shanghai, China), RNA was extracted from pooled HGFs that were stimulated with 1 μg/ml of *P.g* LPS (Invivogen, USA) or unstimulated for 24 hours. For quantitative RT-PCR analyses, the RNA from individual subjects was used.

### miRNA microarray analyses

HGFs were cultured for 4 serial passages in DMEM supplemented with 10% FBS and used in the microarray analyses. HGFs were cultured with DMEM and 10% FBS in the presence or absence of 1 μg/ml of *P.g* LPS (Invivogen, USA) for 24 hours. microRNA profiling analysis was performed by Kangchen Bio-Tech. Briefly, the miRNAs in the RNA samples were labeled using the miRCURY™ Hy3™/Hy5™ Power Labeling Kit (Exiqon, Denmark). The labeled miRNAs were detected by hybridization to miRNA microarrays containing 1769 capture probes (miRCURYTM Array microarray kit, v.11.0, Exiqon) on Bioarray LifterSlip coverslip slides (Genetimes Technology, Shanghai, China). After washing and then drying by centrifugation, the slides were scanned using a Genepix 4000B microarray scanner with a 635 nm laser (Molecular Devices, USA). The fluorescent density data in the images were analysed using Genepix Pro 6.0 software (Molecular Devices) and are presented as the n-fold change of fluorescent density in LPS-stimulated HGFs (LPS+) after normalization with the untreated HGFs (LPS-).

### Reverse transcription

Total RNA was reverse transcribed into cDNA using gene-specific reverse transcription (RT) primers and the MMLV Reverse Transcriptase First Strand cDNA Synthesis Kit (Epicentre, Madison, USA). The RT primers were designed according to the miRNA sequences in the Sanger miRBase. Gene-specific stem-loop primers are listed as follows: hsa-miRNA-146a: GTCGTATCCAGTGC GTGTCGTGGAGTCGGCAATTGCACTGGATACGACaaccca; hsa-miRNA-146b-5p: GTCGTA TCCAGTGCGTGTCGTGGAGTCGGCAATTGCACTGGATAC GACagccta. Each reaction mixture contained 2 μg of DNase-treated total RNA, 50 nM of the RT primers, 1× reaction buffer, 0.25 mM of each dNTP, 200 U of MMLV reverse transcriptase, 20 U of ScriptGuard™ RNase inhibitor, and nuclease-free water (Epicentre) in a total volume of 20 μl. The reaction was performed at 16°C for 30 min, 42°C for 30 min, and 85°C for 5 min in an Applied Biosystems 9700 thermocycler (Applied Biosystems, USA).

### Real-time polymerase chain reaction (PCR)

Real-time PCR was performed as previously described [7]. Briefly, SYBR Green qPCR Master Mix (PA-112, SAbiosciences, Qiagen) was used to detect the levels of miRNA-146a and miRNA-146b-5p on an Applied Biosystems 7900HT Sequence Detection System (Applied Biosystems). The U6 small nuclear RNA (NR_003027) was used as an internal control. Each reaction contained 5 μl of RT SYBR Green qPCR Master Mix, 1.5 μM of the specific forward PCR primer that binds to the 5’ portion of the target miRNA, 0.7 μM of a universal reverse PCR primer that binds to a sequence located in the stem-loop structure, and nuclease-free water in a total volume of 10 μl. The reactions were performed at 95°C for 10 min, followed by 40 cycles of 95°C for 15 s, 60°C for 15 s, 72°C for 15 s, and 55°C for 15 s. All experiments were repeated three times. The sequences of the primers are listed as follows: hsa-miRNA-146a: GGGTGAGAACTGAATTCCA; hsa-miRNA-146b-5p: GGGTGAGAACTGAATTCCA; the universal reverse PCR primer sequence: CAGTGCGTGTCGTGGAGT; U6 small nuclear RNA-forward: GCTTCGGCAGCACATATACTAAAAT and U6-reverse: CGCTTCACGAATTTGCGTGTCAT. The relative expression of each miRNA compared to the U6 small nuclear RNA was calculated using the 2^–△△CT^ method [7].

### miRNA inhibition

miRNA-146 inhibitors were purchased from Applied Biosystems. After 4 passages, the HGFs were transfected with the miRNA inhibitors or a negative control at a concentration of 10 and 100 nM following the manufacturer’s protocol. Opti-MEM (Invitrogen) and Lipofectamine 2000 were purchased from Invitrogen. A total of 10^5^ HGFs were seeded into each well of 24-well plates, incubated overnight and then cultured in Opti-MEM. After 6 hours of transfection with the miRNA and 1 μl of Lipofectamine per well, the medium was replaced with DMEM containing 10% FBS. The supernatant was harvested 18 hours later.

### Flow cytometry

Annexin V and PI stains were used to analyze necrosis and apoptosis, respectively, in HGFs using flow cytometry. Annexin V and PI were purchased from Beyotime (China). The cells were analyzed on a FACSCalibur flow cytometer using CellQuest software (BD Biosciences, USA).

### Enzyme-linked immunosorbent assay (ELISA)

For the analysis of cytokine production in the supernatants of treated and untreated HGFs, human IL-1β, IL-6, IL-10 and TNF-α ELISA Duoset kits were purchased from R&D Systems (USA). The plates were incubated with the appropriate antibodies, aspirated and then washed according to the manufacturer’s protocol. The optical density was detected at 450 nm with a 570 nm compensation.

### Computational predication of the miRNA targets

To further analyze the functions of miRNA-146, we used two computational approaches, MicroRNA.org (http://www.microrna.org) and targetscan (http://www.targetscan.org), to predict the targets of miRNA-146 in the TLR signaling pathways [13,14]. The mirSVR algorithm tool from MicroRNA.org was used to evaluate many features of the identified miRNA targets, including secondary structure-based accessibility of the target site and conservation, without introducing a large number of spurious predictions [14]. The targets that were predicted by both modules and contained acceptable mirSVR down-regulation scores were selected for additional studies.

### Western blot

Immunoblot analyses were performed using SDS-PAGE standard protocols. 10^5^ cells were harvested and lysed (1 mM sodium orthovanadate, 1 mM phenylmethanesulfonylfluoride, 10 μg/ml aprotinin, leupeptin, and pepstatin), with protease inhibitors in the lysis buffer (50 mM HEPES (pH 7.0), 1% Nonidet P-40, 5 mM EDTA, 450 mM NaCl, 10 mM sodium pyrophosphate, and 50 mM NaF). For immunoblot analyses, antibodies against IRAK1 and TRAF6 were obtained from Cell Signaling Technology (USA), and the antibody against β-actin was purchased from Sigma. HRP-labeled secondary antibodies, and super signal west pico chemiluminescent substrate (Pierce, USA) were used to visualize the protein levels.

### Luciferase assay

The IRAK1 3’-UTR sequence (1352 bp starting from TGA of IRAK1) was cloned into the 3’site of the luc2 reporter gene on the pGL4 plasmid (Promega, USA) between the restriction sites *Sal*I and *Bam*HI. We co-transfected 200 ng of the reporter plasmid, 20 ng of pRL-TK-Renilla-luciferase and the miRNA mimic (final concentration 40 nM) in HGFs using Lipofectamine 2000. After 24 hours of transfection, luciferase activity was measured using the Dual-Luciferase Reporter Assay System (Promega) according to the manufacturer’s instructions. The data were normalized for transfection efficiency by dividing firefly luciferase activity with the activity of Renilla luciferase.

### Statistical analysis

The results represent the mean ± standard deviation (SD). Differences in the data were tested for statistical significance using Student’s *t*-test. For all tests, *p* values <0.05 were considered statistically significant.

## Results

### miRNA-146a and miRNA-146b-5p are induced by *P.g* LPS in HGFs

**Figure 1:**
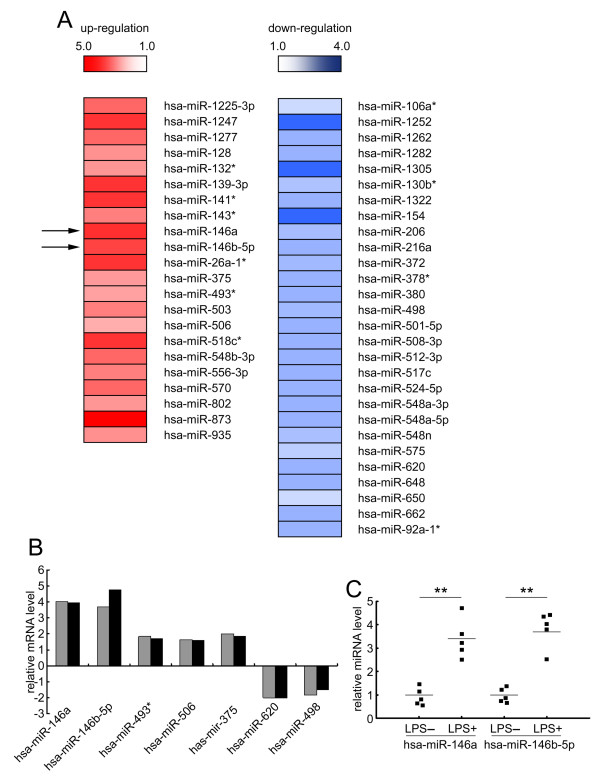
**miRNA-146a and miRNA-146b-5p increase after *P.g *LPS stimulation in HGFs.** (**A**) The fold change of the miRNAs from the miRNA microarray is shown in the schematic diagram. After the HGFs were cultured in the presence or absence of 1 μg/ml of *P.g* LPS for 24 hours, total RNA was collected and used for the miRNA array. The fold change is calculated by dividing the LPS-stimulated samples by the LPS unstimulated normalized samples. The left red bars show the up-regulated miRNAs, and the right blue bars show the down-regulated miRNAs. The colour difference shows the fold change (up-regulation: from 5.0 to 1.0; down-regulation: from 1.0 to 4.0). (**B**) The expression levels of miRNA-146a and miRNA-146b-5p in the pooled RNA samples using real-time PCR. The relative expression levels are presented as the fold change between the LPS-stimulated (24 hour stimulation with 1 μg/ml of *P.g* LPS) and unstimulated HGFs. Black bars refer to microarray results, and grey bars refer to the real-time quantitative RT-PCR results. (**C**) The expression levels of miRNA-146a and miRNA-146b-5p in 5 individual RNA samples using real-time PCR. The relative levels are presented as the fold change between the LPS-stimulated and unstimulated HGFs. The short transverse lines indicate the average values. **: *p* < 0.01.

To explore the miRNA expression patterns in unstimulated and LPS-stimulated HGFs, we performed a miRNA microarray. As shown in Figure 1A, miRNA-146a and miRNA-146b-5p expression levels were increased after *P.g* LPS stimulation. Using real-time PCR, we confirmed 5 up-regulated and 2 down-regulated candidates, which was consistent with the miRNA microarray data (Figure 1B). We found that miRNA-146a and miRNA-146b-5p, which belong to the same miRNA sub-family, showed similar expression patterns. Furthermore, using real-time PCR, we confirmed that miRNA-146a and miRNA-146b-5p increased in pooled RNA samples and five individual RNA samples after *P.g* LPS stimulation (Figure 1C). miRNA-146 up-regulation in *P.g* LPS-stimulated cells suggests that miRNA-146 may regulate the gingival inflammatory response.

### The viability of HGFs after miRNA-146 inhibition

**Figure 2:**
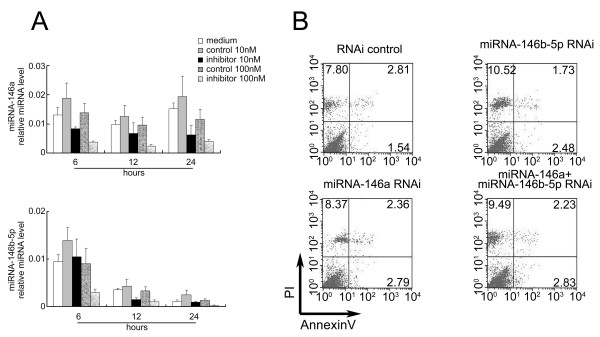
**The viability of HGFs after miRNA-146 inhibition.** (**A**) HGFs were transfected with 10 and 100 nM miRNA-146a and miRNA-146b-5p and stimulated with 1 μg/ml of *P.g* LPS for 24 hours. Total RNA from the HGFs was harvested at different times for the quantitative RT-PCR assays. (**B**) The HGFs were collected and separated by FACS based on PI and Annexin V labeling. The numbers indicate the ratios of necrosis and apoptosis. The results shown represent one of three independent experiments.

To test whether miRNA-146 can affect the response of HGFs to bacteria, we used inhibitors of miRNA-146a and miRNA-146b-5p to knockdown their expression levels in HGFs. The effects of miRNA-146a and miRNA-146b-5p inhibition were measured using real-time PCR (Figure 2A). We found that the levels of both miRNA-146a and miRNA-146b-5p decreased 20-25% when 100 nM of the miRNA inhibitor was used. These results indicate that inhibiting miRNA-146a and miRNA-146b-5p efficiently suppresses miRNA-146a and miRNA-146b-5p levels. We further determined whether the inhibition of miRNA-146 affected necrosis and apoptosis in HGFs. The results indicate that miRNA-146 inhibition has no effect on HGF necrosis and apoptosis (Figure 2B) or proliferation (data not shown). Therefore, miRNA-146 inhibition was used in subsequent experiments to explore the function of miRNA-146 in HGFs.

### Pro-inflammatory cytokine production is increased after miRNA-146 inhibition

**Figure 3:**
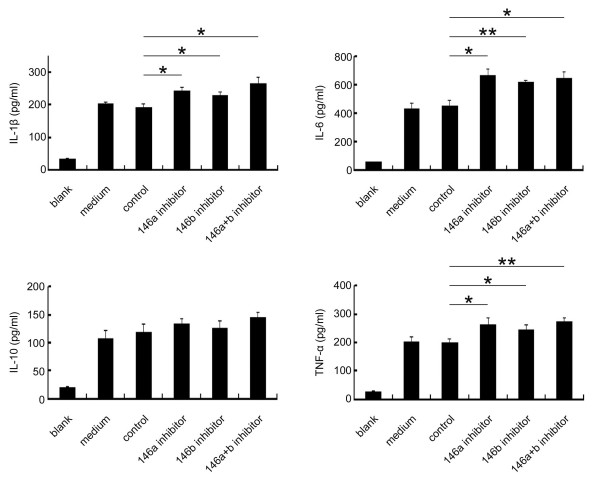
**The inhibition of miRNA-146 increases the production of pro-inflammatory cytokines.** The HGFs were transfected with or without miRNA inhibitors when the cells were confluent. The cells were stimulated with 1 μg/ml of *P.g* LPS for 24 hours, and the cell supernatants were collected for ELISA analysis. The levels of the cytokines are shown as the mean ± SD. *: *p* < 0.05. **: *p* < 0.01. Blank: no LPS treatment; medium: 24 hours stimulation with 1 μg/ml of *P.g* LPS without the miRNA inhibitor; control: 24 hour stimulation with 1 μg/ml of *P.g* LPS after being transfected with a negative control.

In the gingival inflammatory response, cytokines are key regulators of periodontal inflammation [15]. Therefore, we determined if miRNA-146 inhibition could lead to changes in pro-inflammatory cytokine secretion. After miRNA-146 inhibition in HGFs, the production of IL-1β, IL-6, IL-10 and TNF-α was measured (Figure 3). IL-1β, IL-6 and TNF-α production increased after miRNA-146a and/or miRNA-146b-5p inhibition. However, the levels of IL-10, an anti-inflammatory cytokine, were not significantly altered. These data indicate that miRNA-146 can negatively regulate pro-inflammatory cytokine secretion.

### miRNA-146 inhibits cytokine secretion by directly regulating IRAK1 in HGFs

**Figure 4:**
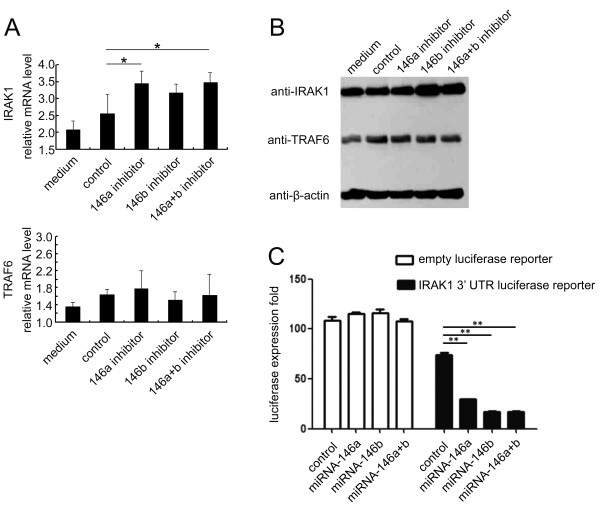
**The attenuation of miRNA-146 enhances Toll-like receptor signaling. A**: HGFs were transfected with a Cy™ 3 labeled dye as a negative control and anti-miRNA-146a/anti-miRNA-146b-5p inhibitors. After 24 hours of stimulation with 1 μg/ml of *P.g* LPS, the expression levels of IRAK1 and TRAF6 were analyzed by real-time PCR (**A**) and immunoblotting (**B**). The immunoblot shown in the figure is representative of three independent experiments. **C**: We amplified the IRAK1 3’-UTR (1352 bp starting from TGA) and cloned the sequence into the 3’ site of the luc2 reporter gene on the pGL4 plasmid. We co-transfected 200 ng of the reporter plasmid, 20 ng of pRL-TK-Renilla-luciferase and indicated miRNA mimic (final concentration 40 nM) into the HGFs. The cells were stimulated with 1 μg/ml of *P.g* LPS after transfection, and the luciferase activity was measured using the Dual-Luciferase Reporter Assay System according to the manufacturer’s instructions. Medium: 24 hour stimulation with 1 μg/ml of *P.g* LPS without the miRNA inhibitor; control: 24 hour stimulation with 1 μg/ml of *P.g* LPS after being transfected with a negative control. The expression levels are shown as the mean ± SD. *: *p* < 0.05.

We next explored the mechanism of miRNA-146-induced pro-inflammatory cytokine secretion. Using computational predications of miRNA targets in the TLR signaling pathways, we found two key factors, IRAK1 and TRAF6, that were potential targets for miRNA-146. To determine if miRNA-146 affected key factors in the TLR pathways, we used miRNA-146a and miRNA-146b-5p inhibition assays in HGFs measured IRAK1 and TRAF6 mRNA and protein levels (Figure 4A and B). The results indicated that the mRNA and protein levels of IRAK1, but not TRAF6, increased. Densitometric analyses of the western blots revealed that transfecting HGFs with miRNA-146a and/or miRNA-146b-5p inhibitors resulted in an increase of IRAK1, but not TRAF6, protein levels (data not shown). These data indicate that miRNA-146 negatively regulates pro-inflammatory cytokine secretion through IRAK1, but not TRAF6. Furthermore, we detected a direct interaction between miRNA146 and the IRAK1 3’-UTR. We cloned the IRAK1 3’-UTR sequence downstream of the luciferase reporter gene. In the luciferase assay, the miRNA-146a and/or miRNA-146b-5p mimics reduced the luciferase levels compared to the control group (Figure 4C). These results suggest that miRNA-146a and miRNA-146b-5p modulate IRAK1 expression by directly targeting the 3’-UTR of IRAK1 mRNA in HGFs. Taken together, miRNA-146 inhibits pro-inflammatory cytokine secretion through IRAK1 in human gingival fibroblasts, which indicates that miRNA-146 functions as a negative regulator in periodontal inflammation.

## Discussion

Although the innate immune response can protect an organism against various pathogens, an excessive or prolonged immune response can be harmful and can result in acute or chronic inflammatory disorders. Thus, the cells involved in the innate immune response must be regulated to inhibit the occurrence of inflammation. It has been demonstrated that miRNA-146 is involved in inflammatory processes [16]. Although the negative regulation of miRNA-146 has been reported in THP-1 monocytes and macrophages [8,10], the effects of miRNA-146 in *P.g* LPS-stimulated HGFs remain unknown.

*P.g* is an important pathogenic organism in human periodontitis, which is a chronic inflammatory disease [15,17,18]. *P.g* LPS is a potent stimulator of inflammatory cytokine production and bone resorption. Lee et al. (2011) compared healthy tissues with tissues infected with periodontitis and found that miRNA-146 was involved in periodontal inflammation [6]. Additionally, in our previous study, we screened the expression of miRNAs in healthy and periodontal-diseased gingiva and found that miRNA-146 expression increased in periodontal-diseased gingiva [7]. In this study, miRNA-146 expression increased in HGFs after stimulation with *P.g* LPS, which further confirmed the previous studies [6,7].

Wang et al. (1999) [15] and Imatani et al. (2001) [19] have shown that HGFs function as regulators of the cytokine network in periodontal tissues and produce inflammatory cytokines in response to the stimulation with bacterial cell components, such as *P.g* LPS. Thus, we further explored whether miRNA-146 regulated the secretion of inflammatory cytokines. After stimulation with *P.g* LPS, HGFs secreted IL-1β, IL-6 and TNF-α, which is consistent with other studies [15,18]. IL-1β, IL-6 and TNF-α are important inducers of inflammation. In the present study, IL-1β, IL-6 and TNF-α levels increased after the inhibition of miRNA-146a and/or miRNA-146b-5p. These data indicate that miRNA-146 negatively regulates the secretion of pro-inflammatory cytokines and prevents aggressive inflammation. However, the mechanism of the miRNA-146-mediated increase in pro-inflammatory cytokines after *P.g* LPS stimulation is still unclear. Nakasa et al. reported that miRNA-146 was induced by TNF-α and IL-1β in the synovial tissues of patients with rheumatoid arthritis [9]. We believe that miRNA-146 could be induced as a result of an increase in TNF-α and IL-1β after stimulation with *P.g* LPS; however, further confirmation is necessary. In this context, miRNA-146 would regulate the immune response in *P.g* LPS-stimulated HGFs.

IL-10 plays a major role in suppressing the immune and inflammatory responses by inhibiting the activity of Th1 cells, NK cells and macrophages [20]. Moreover, Wang et al. [15] reported that LPS-induced IL-6 production was inhibited when HGFs were pretreated with IL-10, suggesting that the anti-inflammatory effects of IL-10 could affect LPS-induced pro-inflammatory cytokine production in vivo. In our study, IL-10 levels increased in *P.g* LPS-stimulated HGFs, indicating that the cells secrete cytokines that negatively regulate inflammation. However, IL-10 levels did not change after the inhibition of miRNA-146a and/or miRNA-146b-5p. The mechanism of IL-10 secretion warrants further investigation.

miRNA-146, like many mammalian miRNAs, may target a wide spectrum of genes . It is noteworthy that miRNA-146 has already been implicated in a number of cellular processes [21-23]. Additionally, it has been demonstrated that miRNA-146 is involved in TLR signaling pathways [9,10]. TLR pathways are involved in innate immunity [24]. Two key adapter molecules in the TLR pathways, TRAF6 and IRAK1, were identified as the target genes of miRNA-146 [10,25]. TRAF6 and IRAK1 yielded high scores on the computational miRNA target prediction algorithms. Our results indicate that miRNA-146a and/or miRNA-146b-5p inhibitors increase IRAK1, but not TRAF6, levels. miRNA-146b-5p had no statistical effect on mRNA level of IRAK1, however, it could negatively regulate translation process of IRAK1, protein level had been tested significantly changed. This indicates miRNA-146b-5p may not involve in IRAK1 binding and mRNA regulation. But based on IRAK1 protein level decreased after miRNA-146b-5p inhibition, it suggests miRNA-146b-5p may regulate another target genes and indirectly affect IRAK1 protein level.

These data indicate that miRNA-146a and miRNA-146b-5p have different effects on IRAK1 expression. miRNAs regulate gene expression by binding to the 3’-UTR of target genes [26]. Our results indicate that miRNA-146a and miRNA-146b-5p bind to the 3’-UTR of IRAK1, suggesting that miRNA-146a and miRNA-146b-5p directly regulate the expression of IRAK1. However, in our study, miRNA-146a and miRNA-146b-5p inhibitors did not result in an increase in TRAF6, which is inconsistent with previous studies [9]. We believe that there are other regulatory mechanisms that control the expression of TRAF6 in HGFs, which is a topic that warrants further investigation. Thus, miRNA-146a and miRNA-146b-5p negatively regulate immune responses by inhibiting IRAK1, but not TRAF6, expression.

## Conclusions

In this study, miRNA-146a and miRNA-146b-5p are up-regulated in *P.g* LPS-stimulated HGFs. The production of pro-inflammatory cytokines, such as IL-1β, IL-6 and TNF-α, increases after miRNA-146 inhibition, and this inhibition is mediated by IRAK1 activation. Additionally, miRNA-146 inhibits IRAK1 expression by binding directly to the 3’-UTR of IRAK1. Our data suggest that miRNA-146a and miRNA-146b-5p may be negative regulators of the immune response by inhibiting IRAK1 expression in periodontal inflammation.

## Abbreviations

miRNA: microRNA; TLRs: Toll-like receptors; IL-1β: Interleukin-1β; IL-6: Interleukin-6; TNF-α: Tumor necrosis factor-α; IRAK1: IL-1 receptor-associated kinase 1; TRAF6: TNF receptor-associated factor 6; HGFs: Human gingival fibroblasts; P.g: *Porphyromonas gingivalis.*

## Competing interests

The authors declare that they have no competing interests.

## Authors’ contributions

RS designed the study, interpreted the results and revised the paper. YX designed the study, participated in cell transfections and flow cytometry, analyzed data and drafted the paper. SJ carried out the RNA extraction, reverse transcription, real-time PCR, ELISA, and western immunoblot. JN cultured the cells. DL and XZ analyzed the data. All authors have read and approved the final manuscript.
